# Post-traumatic stress disorder and body satisfaction among patients at Ruhigita clinic, Bukavu (DRC): an observational study

**DOI:** 10.3389/fpsyg.2025.1704684

**Published:** 2025-12-11

**Authors:** Justin Cikuru, Philippe Kaganda, Adélaïde Blavier, Jennifer Foucart

**Affiliations:** 1 Health Psychology and Human Movement Sciences Research Unit, University of Brussels (U.L.B), Brussels, Belgium; 2 Faculty of Social Sciences, Evangelical University of Africa (U.E.A), Bukavu, Democratic Republic of Congo; 3 Faculty of Psychology, Speech Therapy and Educational Sciences, Center of Expertise in Psychotrauma and Forensic Psychology, University of Liège, Liège, Belgium

**Keywords:** PTSD, body satisfaction, somatic symptom, Ruhigita clinic, Bukavu

## Abstract

**Background:**

Over the past two decades, armed conflicts have intensified globally, with Africa disproportionately affected. Since January 2025, renewed violence by the March 23 Movement (M23) in eastern Democratic Republic of Congo (DRC) has generated widespread trauma, displacement, and psychological distress. Beyond emotional suffering, trauma has been linked to altered body perception and reduced body satisfaction, particularly among women survivors of violence, adding further complexity to the psychological burden.

**Objectives:**

This study assessed the prevalence of post-traumatic stress disorder (PTSD) among hospital patients in Bukavu and examined associations with body-related symptoms and body satisfaction. It was hypothesized that many patients would meet PTSD criteria, that affected individuals would report lower body satisfaction and greater body-related distress, and that only a small minority would have accessed psychological care.

**Methods and materials:**

Data were collected at Ruhigita Clinic, South Kivu. Adults aged 18–65 completed the PTSD Checklist (PCL-5, French version) and the Bruchon-Schweitzer Body Satisfaction Questionnaire. A PTSD score ≥32 indicated clinical symptoms; adequate body satisfaction was defined as ≥3. Interviews lasted 45–60 min and included demographic and trauma-related data.

**Results:**

A total of 356 patients participated. The mean PTSD score (*M* = 23.49; SD = 19.90) was below the diagnostic threshold; however, 31.5% (*n* = 112) met PTSD criteria. Among them, 57.1% reported dissatisfaction with body appearance, compared to 32.4% of non-PTSD participants. PTSD was significantly associated with somatic symptoms such as hypertension, diabetes, stomach pain, insomnia, and cardiac complaints. Reported traumas included natural disasters (74.6%), interpersonal violence (73.7%), transport accidents (54.8%), and sexual assaults (54.1%). Natural disasters, particularly floods and wildfires, showed strong associations with PTSD onset. Despite 80% awareness of psychological services, only 9.8% had consulted a clinical psychologist. Gender differences emerged: women relied mainly on religious or spiritual support, while men favoured traditional practices.

**Conclusion:**

This study confirms a strong link between PTSD, body dissatisfaction, and somatic symptoms in a conflict-affected population. Despite high awareness of distress, mental health service use remains low. Findings highlight the need for integrative, culturally sensitive interventions that respect local understandings of trauma and healing while addressing urgent gaps in psychological care.

## 1 Introduction

Armed conflicts have escalated worldwide over the past two decades, with Africa being the most affected continent (Hortence, 2022). Democratic Republic of the Congo (DRC), and specifically its eastern region, has endured decades of armed conflict since the aftermath of the Rwandan genocide (Ba, 2024). Often described as one of the world’s most neglected crises, the situation in eastern DRC has resulted in devastating humanitarian consequences, including over 7 million displaced people (Zina et al., 2024). The violence marked by sexual assault, mass killings, and widespread destruction has left lasting trauma across generations (Mehinto et al., 2024). According to Florentin and Josué (2024), the recurring cycles of war (1996, 1998, 2008), and the ongoing M23 rebellion have deeply scarred the population, leading to long-term psychological distress (Alexandre et al., 2025a,b; Hatem et al., 2025).

Since January 23, 2025, the March 23 Movement (M23) has intensified violence in eastern DRC, leading to loss of life, displacement, sexual violence against women and children and destruction of infrastructure in Bukavu (Hatem et al., 2025). Trauma causes more deaths in Sub-Saharan Africa than tuberculosis, HIV, malaria, and COVID-19 combined, accounting for over 90% of global injury-related fatalities (Osebo et al., 2025). This disproportionate burden is linked to poor infrastructure, a lack of trauma centers, limited medical resources, and a shortage of trained healthcare workers (Reynolds et al., 2017). Trauma from armed conflict, natural disasters, violence, and road accidents also contributes significantly to morbidity and accounts for 6% of global burden of years lived with physical and mental disability such as Post-Traumatic Stress Disorder (PTSD) (Bundu et al., 2019; Gebreyesus et al., 2024).

PTSD is a psychiatric condition that may develop following exposure to one or more traumatic events and is characterized by intrusive memories, avoidance, negative alterations in cognition or mood, and persistent hyperarousal (American Psychiatric Association et al., 2016). This psychological disease is defined as a condition that occurs after exposure to traumatic events, characterized by symptoms such as re-experiencing, hyperarousal, and emotional or cognitive disturbances (Zina et al., 2024). Literature suggests that 10% to 20% of individuals exposed to trauma may develop PTSD (Watkins et al., 2018). The pooled prevalence of PTSD in Sub-Saharan Africa is estimated at 22% (Kaminer et al., 2024), with even higher rates around 29% reported in countries affected by war and conflict, particularly in low- and middle-income countries (Lim et al., 2022). In this context victims are often first taken to local community or rural health facilities rather than designated trauma centers (McIver et al., 2024). In Bukavu, one of the region heavily affected by conflict, the true prevalence of PTSD remains unknown due to limited documentation (Lim et al., 2022). Studies have also established a link between PTSD and decreased body satisfaction, a central dimension of body image referring to an individual’s subjective evaluation of their physical appearance (Kaminer et al., 2024). Negative body satisfaction is consistently associated with trauma exposure and adverse mental health outcomes, including PTSD. Recent findings by Gopan et al. (2024) reinforce evidence that PTSD is closely linked to decreased body satisfaction, a pattern also reported by Kaminer et al. (2024). Gopan et al. (2024) found that PTSD survivors frequently report dissatisfaction with their body image. From a psychological perspective, Moussa et al. (2024) note that individuals who lose a limb due to war or accidents often experience denial, a reaction that interferes with the mourning process and the mobilization of adaptive defence mechanisms. This denial can lead to sleep and anxiety disorders, diminish quality of life through functional limitations and social withdrawal, and ultimately result in regressive psychological functioning. Despite these insights, significant gaps remain. Most research on PTSD and body satisfaction has been conducted in high-income countries or specialized populations (e.g., veterans, burn survivors, university students) as highlighted by Rupani et al. (2024). Very few studies examine these associations in routine hospital populations in conflict-affected African settings. Moreover, almost no studies have explored PTSD, body satisfaction, and help-seeking behaviours simultaneously.

In eastern DRC, individuals affected by violence frequently seek help outside formal mental health services, relying on religious or traditional healing, and only a minority consult mental health professionals (Balegamire, 2021; Mehinto et al., 2024). Clinical observations at Panzi Hospital highlight the persistence of psychological distress even after treatment for physical injuries, particularly among survivors of sexual violence facing PTSD, intergenerational trauma, gynaecological complications, stigma, and social shame (Mukwege and Berg, 2016; Zina et al., 2024).

Psychosocial services embedded within a holistic model of care have been developed to address these complex needs, including counseling, music therapy, dance therapy, occupational therapy, and cognitive-behavioural therapy (Alexandre et al., 2025a,b; Cikuru et al., 2021; Mukwege and Berg, 2016). However implementation across all hospitals in Bukavu remains challenging due to staffing shortages, limited training, and role confusion (Florentin and Josué, 2024; Raslan et al., 2021). Strengthening mental health systems and building a culturally grounded workforce remains an urgent priority (Tol et al., 2020).

However, providing psychosocial support within integrated health services for survivors of trauma in all the hospitals in Bukavu is not that straightforward in a complex humanitarian context (Florentin and Josué, 2024). Despite the under-recognition of psychosocial care to survivors of wartime sexual violence, like other conflict-affected settings, the shortage of trained psychosocial workers leads to task overload and role confusion among, many of whom the lack of formal and cultural base training in psychosocial support often substituted with nurses (Raslan et al., 2021). In post-conflict settings where mental health issues are prevalent there is a pressing need for stronger policies and well-trained mental health practitioners (Tol et al., 2020). This study aims to estimate the prevalence of PTSD among patients at Ruhigita Clinic in Bukavu, South Kivu (DRC), examine its relationship with body satisfaction, and assess patterns of help-seeking behavior. Objectives are threefold: estimate PTSD prevalence, examine the relationship between PTSD and body satisfaction, and assess the proportion of patients seeking psychological support.

## 2 Methods and materials

### 2.1 Study design and setting

As noted by Tasu et al. (2024), descriptive studies help identify trends, frequencies, and associations without implying causality, whereas analytical studies aim to explore relationships between variables and assess potential causal links. This research was conducted as an observational study, using an analytical cross-sectional design within a quantitative approach, enabling assessment of associations between independent factors such as trauma exposure and outcome variables (PTSD and body satisfaction).

The study was conducted at Clinique Ruhigita, a private healthcare facility located in the Ibanda Health Zone of Bukavu, South Kivu, DRC. This region has a population of approximately 496,020 residents (Sivyavugha et al., 2024). The clinic provides outpatient services to the local community and serves as a referral point for patients from nearby health zones (Kadutu, Ibanda, Bagira). Data were collected from February 4, 2024, to March 4, 2025, using face-to-face interviews that lasted 45–60 min.

### 2.2 Study population and eligibility

The target sample included adults aged 18 to 65 years attending the outpatient department of the hospital. Participants were required to be fluent in French and/or Swahili and to provide written informed consent. Patients presenting for routine follow-up or laboratory tests not related to a medical consultation were excluded to ensure relevance to trauma exposure and psychological assessment. The sampling formula yielded a target of 384 participants. However, 28 individuals could not be included due to refusal to participate, incomplete interviews, inability to provide informed consent, or time constraints, resulting in a final sample of 356 participants.

### 2.3 Sample size determination and sampling technique

A non-proportional stratified random sampling method was employed (Fleetwood, 2018), stratified by the health zones of Bukavu: Kadutu, Ibanda, and Bagira. The sample size was calculated using the single population proportion formula:


$$N=\frac{Z_{1-\alpha /2}^{2}\cdot P(1-P)}{d^{2}}\gamma ?$$

Where:

-
$Z_{1-\alpha /2}=1.96$ (for 95% confidence level).
-
P=0.50 was selected because local prevalence data for PTSD and body satisfaction in Bukavu are limited, providing the most conservative estimate.
-
d=0.05 (margin of error).

The calculated sample size was 384 participants. Considering non-response (~7.3%, 28 individuals), the final achieved sample was 356 participants. The non-response was attributed to refusal to participate, time constraints, and the inability to complete the interview.

### 2.4 Data collection tools

1. Two validated instruments were used: The Post-Traumatic Stress Disorder Checklist - PCL-5 (Ashbaugh et al., 2016), based on the DSM-5, this tool assesses exposure to traumatic events and PTSD symptoms. A score ≥32 was used to indicate clinically significant PTSD.
2. The Bruchon-Schweitzer Body Image Questionnaire (BIQ) (1990), assessing body satisfaction on a 1–5 scale. A mean score ≥3 indicates adequate body satisfaction (Koumba et al., 2024; Moussa et al., 2024). The instrument was not modified, but clarifications were provided during administration to ensure patient understanding.

### 2.5 Data analysis

Data were collected using KoboToolbox, cleaned with Excel, and analysed using SPSS v29.0.2. Descriptive statistics (frequencies, percentages, means, and standard deviations) were computed. Bivariate and multivariate analyses (including Pearson correlation and regression analyses) were performed to assess associations between PTSD, body satisfaction, and independent demographic and clinical factors.

### 2.6 Ethical clearance

Ethical clearance was obtained from National Health Ethics Committee, South Kivu Provincial Board CNES, standing for (Comité National d’Ethic de la santé), Ref No.: CNES/DP-SK001/4125/001–2024. Participants of the study were communicated, and written consent was obtained before the data collection. To protect the participants, no individual identifying information was collected. Respondents were informed of their right to withdraw from the study at any time with no subsequent harm for refusal of participation. All methods were carried out by relevant guidelines and regulations of CNES.

## 3 Results

### 3.1 Sociodemographic distribution in the sample

There were 356 participants out of 384 who participated in the study, yielding a response rate of 92.7%. The average age of participants was 40 years (SD = 12), indicating a predominance of middle-aged adults (31–50 years), with a relatively balanced distribution across age groups. A majority of women were observed across all age categories. This trend was particularly pronounced among young adults (73%) but remained significant among middle-aged adults (68.1%) and seniors (57.3%), where the gap with men narrows. Regarding educational attainment, women predominated at the primary, secondary, and university levels. However, vocational training showed a reversal of this trend, with more men represented. The distribution by marital status reflects the same preponderance of women: whether single (70%), living with a partner (65.8%), alone with children (65.8%), or alone without children (69%).

### 3.2 Predominance of PTSD in the sample

#### 3.2.1 PTSD score statistics with a cut-off ≥32

**Figure 1:**
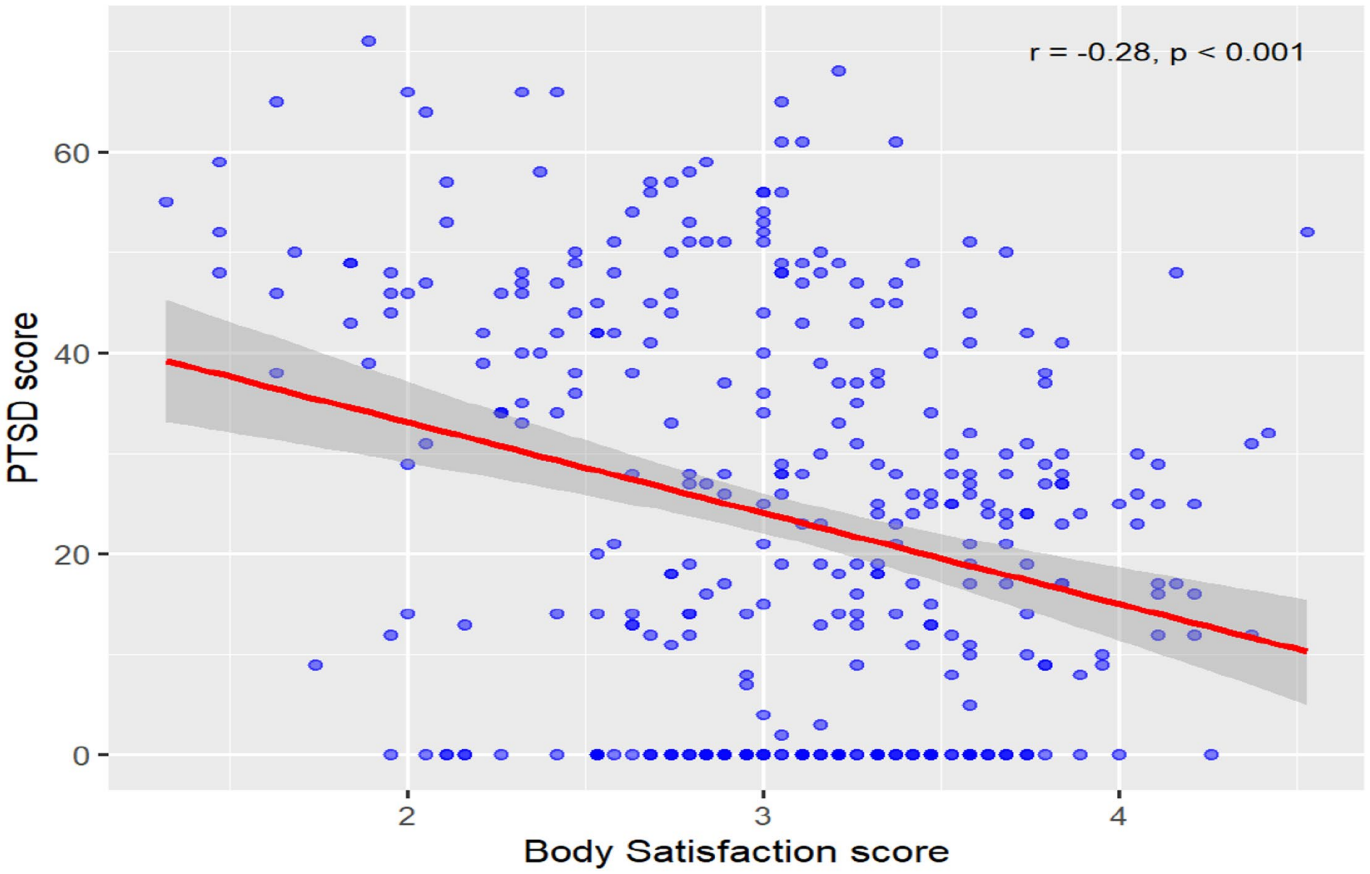
Correlation between body satisfaction and PTSD.

From a total of 356 participants who took part in this study, 112 (31.5%) met the criteria for PTSD, while 244 (68.5%) did not. These findings support our first hypothesis, which predicted that more than 10% of patients consulting hospitals in Bukavu would have PTSD. In our sample of 238 women, 78 (32.8%) met the criteria for PTSD. Among 118 men, 34 (28.8%) had PTSD. Although the proportion of women with PTSD was slightly higher than that of men, this difference was not statistically significant at the 0.05 level, (*χ*^2^ = 0.574^a^, df = 1, sig = 0.449), indicating that PTSD prevalence did not differ meaningfully between men and women in this sample. Among the potentially traumatic events reported by participants, we found natural disasters and fires constituted the most frequent category, affecting 74.6% of the sample. Interpersonal violence, including physical assaults and armed assaults (related to wartime contexts), also affected many participants at 73.7%. Transport accidents and other related traumatic situations affected 54.8% of the sample. Finally, sexual assaults and other non-consensual sexual experiences were reported at 54.1% of the total (see Figure 1).

#### 3.2.2 Medical consultation reasons

This section highlights the symptoms presented by the 112 participants having PTSD (Table 1), as their reasons for medical consultation. It seeks to determine whether there is a statistically significant relationship between the somatic symptoms of the participants and the pathological threshold of post-traumatic stress disorder (PTSD), according to the reason for consultation. The multiple linear regression model (Table 2) revealed that specific physical health symptoms significantly predicted PTSD symptom severity. In particular, hyper/hypotension (*β* = 0.445, *p* < 0.001), cardiac symptoms such as tachycardia and respiratory problems (*β* = 0.225, *p* < 0.001), and somatic complaints (*β* = 0.253, *p* = 0.049) were associated with higher PTSD scores. Conversely, diabetes was significantly associated with lower PTSD symptoms (*β* = −0.142, *p* = 0.010). Other physical health indicators, including digestive issues, headaches, sleep disturbances, and sexual infections, did not reach statistical significance.

**Table 1:** PTSD and somatic symptoms.

Multiple linear regression model^a^	Unstandardized coefficients		Standardized coefficients	*t*	Sig.
(Constante)	18.122	1.621		11.181	<0.001
Somatic pain (Dizziness, back pain, fever and flu, cough and fainting)	10.086	5.101	0.253	1.977	0.049
Digestive pain (abdominal pain, nausea, diarrhea or constipation)	−7.552	5.094	−0.188	−1.483	0.139
Hyper/hypo blood pressure	23.794	3.464	0.445	6.869	<0.001
Diabetes	−15.009	5.801	−0.142	−2.587	0.010
Digestive problems	−1.580	3.968	−0.033	−0.398	0.691
Chronic Headaches	−4.015	3.495	−0.085	−1.149	0.251
Difficulty sleeping	−0.234	1.931	−0.006	−0.121	0.904
Sexual infections	−6.212	13.392	−0.068	−0.464	0.643
Cardiac symptoms (Tachycardia, palpitations and breathing problems)	12.475	2.703	0.225	4.615	<0.001
Skin rash	5.837	14.059	0.061	0.415	0.678

a Dependent variable: PTSD score.

**Table 2:** Body satisfaction score and somatic symptoms.

Multiple linear regression model^a^	Unstandardized coefficients		Standardized coefficients	t	Sig.
(Constante)	3.210	0.055		58.149	<0.001
Somatic pain (Dizziness, back pain, fever and flu, cough and fainting)	−0.032	0.174	−0.027	−0.186	0.852
Digestive pain (abdominal pain, nausea, diarrhea or constipation)	−0.140	0.173	−0.114	−0.808	0.420
Hyper/hypo blood pressure	−0.313	0.118	−0.191	−2.653	0.008
Diabeties	0.210	0.198	0.065	1.061	0.289
Digestive problems	0.130	0.135	0.087	0.960	0.338
Chronic Headaches	−0.080	0.119	−0.055	−0.671	0.502
Difficulty sleeping	−0.004	0.066	−0.003	−0.061	0.952
Sexual infections	−0.687	0.456	−0.247	−1.506	0.133
Cardiac symptoms (Tachycardia, palpitations and breathing problems)	−0.243	0.092	−0.143	−2.642	0.009
Skin rash	0.849	0.479	0.289	1.773	0.077

a Dependent variable: body satisfaction score.

When we compare the body satisfaction and somatic symptoms, the regression analysis examining predictors of body satisfaction (Table 2) revealed that blood pressure issues (*β* = −0.191, *p* = 0.008) and cardiac-related symptoms (*β* = −0.143, *p* = 0.009) were significantly associated with lower levels of body satisfaction. These findings suggest that individuals experiencing cardiovascular symptoms may perceive their bodies more negatively. While most other physical health conditions, including digestive complaints, diabetes, chronic headaches, and sleep difficulties did not significantly predict body satisfaction, skin rash presented a marginally significant positive association (*p* = 0.077).

Pearson’s correlation analysis revealed a statistically significant negative association between body satisfaction and PTSD symptom severity (*r* = −0.277, *p* < 0.001), indicating that individuals with higher levels of body satisfaction tend to report lower levels of post-traumatic stress symptoms. Although the correlation was modest in magnitude, it remains statistically robust and suggests that body image may play a meaningful role in the psychological well-being of individuals affected by trauma. In the multiple regression analysis, physical health symptoms, particularly blood pressure irregularities (*β* = −0.191, *p* = 0.008) and cardiac-related symptoms (*β* = −0.143, *p* = 0.009), were significantly associated with lower levels of body satisfaction. These results suggest that cardiovascular symptoms may negatively influence body satisfaction. In contrast, most other physical health indicators, including digestive complaints, chronic headaches, and sleep disturbances, did not demonstrate significant predictive value.

### 3.3 Body satisfaction and post-traumatic stress disorder

The analysis of body satisfaction used a cut-off score of ≥3, considered as an indicator of body satisfaction (Koumba et al., 2024). We found that the mean score of 3.0638 suggests that, overall, most participants have a relatively positive perception of their bodies. However, the moderate variability (standard deviation of 0.60976) indicates that some participants scored well below this threshold, pointing to marked body dissatisfaction. The minimum score of 1.32 suggests that certain individuals experience significant discomfort with their bodies. Therefore, we proceeded to analyse the distribution of scores to determine the exact proportion of participants falling below the body satisfaction threshold.

The relationship between participants’ gender and their level of body satisfaction (Table 3) shows that, out of the 356 participants in the sample, 60.1% of women and 59.3% of men report being satisfied with their bodies, while 39.9% of women and 40.7% of men report being dissatisfied. These proportions are very similar between the two genders. To determine whether this distribution is statistically significant, a chi-square test of independence was performed. The results indicate that there is no significant relationship between gender and body satisfaction, *χ*^2^ (1, *N* = 356) = 0.019, *p* = 0.890. Fisher’s exact test confirms this absence of association (*p* = 0.909). Thus, gender does not appear to significantly influence body satisfaction within our sample, in which 40.2% have a negative perception of their body. This led us to analyse the group that would be more affected compared to the pathological threshold of PTSD.

**Table 3:** Cross-tabulation of participant gender and body satisfaction threshold.

Gender	Not satisfied with his body	Satisfied with his body	Total
Women	95^a^	143^a^	238
	39.9%	60.1%	100%
Man	48^a^	70^a^	118
	40.7%	59.3%	100%
Total	143	213	356
	40.2%	59.8%	100%

*χ*^2^ = 0.019^a^, df = 1, sig = 0.890, Fisher’s exact test (*p* = 0.909) a. 0 cells (0.0%) have a theoretical number less than 5.

Overall data presented in Table 4 show that 40.2% of participants report being dissatisfied with their bodies, while 59.8% express body satisfaction. Among patients without PTSD (*N* = 234), a clear majority (67.6%) report being satisfied with their bodies. In contrast, among individuals with PTSD (*N* = 112), this trend is reversed, 57.1% report being dissatisfied with their body image. These figures indicate a marked difference in body perception depending on the presence of PTSD (see Figure 1).

**Table 4:** Cross-tabulation of participant gender and PTSD threshold.

	Not satisfied with his body	Satisfied with his body	Total
No PTSD	79^a^	165^a^	244
	32.4%	67.6%	100%
Has PTSD	64^a^	48^b^	112
	57.1%	42.9%	100%
Total	143	213	356
	40.2%	59.8%	100%

*χ^2^* = 22.870^a^, df = 1, sig = *p* < 0,001, Yates continuity correction (21.794, *p* < 0.001), likelihood ratio (22.733, *p* < 0.001), Fisher’s exact test (*p* < 0.001), linear association test (22.806, *p* < 0.001).

“^b^” indicates that this proportion is different from the other category in the same row.

### 3.4 Correlation between body satisfaction and PTSD

Statistically, the association between the two variables (Table 5) is confirmed by highly significant results. Pearson’s correlation analysis revealed a significant negative association between body satisfaction and PTSD symptom severity (*r* = −0.277, *p* < 0.001, *N* = 356). This result indicates that higher levels of body satisfaction are significantly associated with lower levels of post-traumatic stress symptoms. In line with the hypothesis that individuals with PTSD would show lower body satisfaction, a Pearson correlation analysis revealed a significant negative correlation, (*r*) = −0.277**, *p* < 0.001. This result indicates that a higher level of PTSD symptoms is associated with a lower level of body satisfaction. Although the strength of the association is moderate, its statistical significance indicates the presence of a weak but meaningful relationship between PTSD and body satisfaction: the more severely a patient is affected by PTSD, the lower their body satisfaction tends to be.

**Table 5:** Correlation between body satisfaction and PTSD.

Correlations		Body satisfaction score	PTSD score
Body Satisfaction score	Correlation de Pearson	1	−0.277^a^
	Sig. (two-tailed)		<0.001
	*N*	356	356
PTSD score	Correlation de Pearson	−0.277^a^	1
	Sig. (two-tailed)	<0.001	
	*N*	356	356

a The correlation is significant at the 0.01 level (two-tailed).

Chi-square was used to assess the association between the type of consultation and (i) PTSD and (ii) body satisfaction. the “^a^” indicates that the reported Ch-square values correspond to these tests.

### 3.5 Access to psychological services and use of traditional or spiritual alternatives

Data analysis reveals that most participants (80.4%) report being informed about the existence of clinical psychology services, with a slight gender variation: 83.3% of women versus 73.5% of men. However, this difference does not appear to be statistically significant. Despite the high level of information, only 9.8% of respondents report having consulted a clinical psychologist, that is, 11 participants out of a total of 112. This low proportion, similar among women (10.3%) and men (8.8%), supports the hypothesis that less than 50% of individuals exhibiting PTSD symptoms seek clinical psychological support. The data highlight substantial use of alternative forms of help. More than half of the participants (51.8%) report having consulted a traditional healer, with a higher proportion among men (64.7%) than women (53.8%). Regarding spiritual support, 50% of participants report belonging to a prayer group. Notable gender differences emerge here as well, 59% of women participate in such groups compared to only 29.4% of men. Thus, although clinical psychology services are generally recognized, their utilization rate remains low within the sample. The findings highlight a preference for alternative forms of support, with gender-based differences, women tend to favour religious spiritual support, while men are more inclined toward individualized traditional approaches.

The finding that over half of participants seek help from traditional healers or prayer groups reflects a persistent pattern of medical pluralism in sub-Saharan Africa, where around 50% of individuals with mental health concerns consult traditional or faith-based healers before biomedical professionals. This preference is shaped by accessibility, cultural embeddedness, and the ability to address both spiritual and social dimensions of distress (Omigbodun et al., 2023). Gender differences also align with broader anthropological insights. Men tend to favour individualized, ritual-based healing such as herbal treatments or ancestral rites, driven by ideals of masculinity and self-reliance that discourage engagement with formal mental health services (Sikweyiya et al., 2025). In contrast, women often turn to collective religious spaces like prayer groups, which provide emotional support and culturally legitimate avenues for expressing suffering. In the Est of DRC, these help-seeking behaviours correspond with local understandings of trauma as spiritual misfortune or harm caused by others, best treated through ritual or prayer. Clinical psychology, with its abstract diagnostic language such as PTSD, may appear incomplete or culturally distant if it is not grounded in local contextual practices.

### 3.6 Consultation types, PTSD and body satisfaction

The cross-analysis of the types of consultations undertaken by participants with levels of post-traumatic stress disorder (PTSD) and body satisfaction (Table 6) reveals associations between the variables, although these are not statistically significant. Participants who consulted only a medical doctor (*n* = 69) showed the lowest proportion of PTSD (21.7%). In contrast, PTSD rates increase among participants who engaged in combined consultations, particularly those involving pastors, traditional healers, or psychologists. For example, 40.7% of participants who consulted a doctor, a pastor, and a traditional healer had PTSD; this proportion rises to 50% for those who consulted both a doctor and a psychologist, and up to 60% for those who consulted a doctor, a pastor, and a psychologist.

**Table 6:** Association between consultation types, PTSD and body satisfaction.

Multiple consultations	Post-traumatic stress disorder			Body satisfaction		
	No PTSD	Has PTSD	Total	Not satisfied with his body	Satisfied with his body	Total
Consultation Med oly	54	15	69	16	53	69
	78.3%	21.7%	100%	23.2%	76.8%	100%
Consultation Med-Past-trad	48	33	81	37	44	81
	59.3%	40.7%	100%	45.7%	54.3%	100%
Consultation Med-Past	90	36	126	56	70	126
	71.4%	28.6%	100%	44.4%	55.6%	100%
Consultation Med-Trad	40	18	58	25	33	58
	69%	31%	100%	43.1%	56.9%	100%
Consultation Med-Psy	4	4	8	3	5	8
	50%	50%	100%	37.5%	62.5%	100%
Consultation Med-Past-Psy	2	3	5	2	3	5
	40%	60%	100%	40%	60%	100%
Consultation Med-Trad-Psy	6	3	9	4	5	9
	66.7%	33.3%	100%	44.4%	55.6%	100%

(*χ*^2^ = 9.930^a^, df 6, *p* = 0.128) for PTSD and (*χ*^2^ = 10.560^a^, df 6, *p* = 0.103) for body satisfaction.

These findings suggest that individuals with PTSD often seek multiple forms of care, particularly incorporating spiritual and traditional support systems. This pattern of help-seeking behavior may be attributed to unmet needs within formal medical services, including the lack of psychological infrastructure and limited access to qualified professionals (Omigbodun et al., 2023; Gopan et al., 2024).

A similar trend appears in relation to body dissatisfaction, where the distress is not only experienced psychologically but interpreted and addressed through local cultural and spiritual frameworks. In Bukavu, the predominance of Christian religious beliefs, particularly Pentecostalism and Catholicism, plays a crucial role in shaping how individuals understand and manage suffering. Spiritual healing, often through prayer groups or deliverance rituals, is viewed as a legitimate and sometimes preferred avenue for addressing trauma and bodily distress (Mehinto et al., 2024; Sikweyiya et al., 2025). Moreover, the economic hardship faced by much of the population limits access to formal health services, reinforcing reliance on magico-religious practices in contexts where biomedicine fails to offer relief. These practices are deeply embedded in local believe that view illness as the result of spiritual forces, ancestral disharmony, or social conflict (Omigbodun et al., 2023).

Among participants who only consulted a doctor, 76.8% reported being satisfied with their bodies, compared to lower satisfaction rates in groups that accessed multiple types of consultations, 54.3% in the “Med-Past-Trad” group, 55.6% in the “Med-Past” group, and 56.9% in the “Med-Trad” group. Overall, participants with both PTSD and body dissatisfaction are more frequently found among those who engaged in multiple types of consultations. This finding suggests that multiple consultations may serve as an indirect indicator of psychological vulnerability, as medical-only consultations are associated with higher body satisfaction, while combined approaches are more common among individuals who are dissatisfied with their bodies and suffer from PTSD.

## 4 Discussion

The purpose of this research was to analyse the predominance of post-traumatic stress disorder (PTSD) among patients consulting medical service at Ruhigita hospital and explore its relationship with body satisfaction among the participants. The results obtained reveal that, of the 356 participants, 112 (31.5%) had PTSD. Among the 238 women in our sample, 78 (32.8%) met criteria for PTSD. Among the 118 men, 34 (28.8%) had PTSD. In the remaining participants, the proportion without PTSD was comparatively higher.

These results are consistent with current literature and slightly exceed PTSD rates reported in previous studies, which documented prevalences up to 29% in war-affected regions of sub-Saharan Africa (Lim et al., 2022). This correspondence with previous research was assessed by comparing regional studies conducted among African populations affected by conflict, using similar diagnostic tools (PCL-5) and identical PTSD thresholds (≥32). This higher prevalence in our study is understandable given the region’s history of armed conflict, sexual violence, and chronic insecurity (Tesfaye et al., 2024). When analysing by gender, women appeared slightly more affected (32.8%) than men (28.8%), although this difference was not statistically significant. Nevertheless, this trend aligns with the literature indicating that women may be more vulnerable to PTSD due to a combination of biological, psychological, and sociocultural factors, as well as their greater exposure to rape and sexual or gender-based violence (Olff, 2017). The comparatively lower prevalence among men may be explained by underreporting, cultural norms discouraging the expression of psychological distress, or different exposure patterns (e.g., less sexual violence) in conflict contexts. A significant relationship between PTSD and body satisfaction was observed. Among participants with PTSD (*N* = 112), 57.1% reported body dissatisfaction, compared to 32.4% among those without PTSD. Overall, 60.1% of women and 59.3% of men reported being satisfied with their bodies, while 39.9% of women and 40.7% of men reported dissatisfaction. This pattern reflects established psychological mechanisms whereby trauma, particularly interpersonal trauma such as sexual violence, can disrupt body perception, leading to increased self-criticism, dissociation, and negative self-image (Andualem et al., 2024; Momeñe et al., 2023). These results support the hypothesis that PTSD is not only a condition characterized by emotional and cognitive symptoms, but also significantly affects somatic aspects and body satisfaction (Gopan et al., 2024).

The study also highlights association between post-traumatic stress disorder (PTSD) and various somatic symptoms, such as hypertension, diabetes, stomach pain, insomnia, and cardiac disorders. These findings are consistent with recent research indicating that individuals with PTSD frequently experience a range of physical health issues. A systematic reviews showing that chronic somatic conditions and PTSD are interconnected, suggesting a bidirectional relationship (Lunkenheimer et al., 2023; Jowett et al., 2020). Conversely, our study did not identify significant associations between PTSD and certain symptoms such as digestive pain, headaches, skin rashes, and sexually transmitted infections. The variation in associations depending on symptom type may be explained by symptom-specific vulnerability and differences in reporting, consistent with Wright et al. (2021).

Regarding trauma exposure, natural disasters such as floods and wildfires have been increasingly linked to the onset of post-traumatic stress disorder (PTSD). Participants reported multiple trauma types: natural disasters (74.6%), interpersonal violence (73.7%), transport accidents (54.8%), and sexual assaults (54.1%). This cumulative exposure is consistent with findings from humanitarian contexts, showing that repeated or multiple traumas significantly increase PTSD risk (Caldiroli et al., 2022). Recent studies also highlight the psychological impact of climate-related disasters; for example, survivors of wildfires often exhibit significantly elevated PTSD, anxiety, and depression symptoms even months after the event (Jiang et al., 2021).

Although clinical psychology services are generally recognized in the region, their utilization remained low: only 9.8% of participants aware of these services had consulted a psychologist. Gender differences were observed: women preferred religious or spiritual support, whereas men favored traditional approaches. Barriers to mental-health service use in sub-Saharan Africa include financial constraints, structural limitations, cultural and linguistic barriers, and stigma (Galvin et al., 2024; Van der Zeijst et al., 2023; Pigeon-Gagné et al., 2022; Gbagbo, 2024). Additionally, lack of awareness and shortages of trained professionals further limit access to care (Kounou et al., 2020).

## 5 Study limitations, strengths, and perspectives

This study presents methodological limitations that should be considered when interpreting its findings. The cross-sectional design limits causal inference, restricting conclusions to correlational relationships between post-traumatic stress disorder (PTSD), body satisfaction, and care-seeking behavior. The research was conducted in a single private healthcare facility within the Ibanda Health Zone of Bukavu, which limits the generalizability of the results to other clinical, geographical, or sociocultural contexts. However, the sample of 356 participants, determined using a statistical formula for estimating proportions, provides a sufficient basis for consideration in this study.

Potential selection bias may have been introduced by the inclusion criteria, which required participants to be fluent in French or Swahili and willing to engage in a structured one-hour interview. Although the researcher was present to provide clarification during data collection, individuals with limited literacy or language skills may have been inadvertently excluded. The exclusive reliance on self-reported data also raises concerns about recall bias and social desirability effects. Moreover, the study did not account for potential confounding variables, such as comorbid psychiatric conditions, which may have influenced the strength and direction of the observed associations.

Despite these limitations, this study makes a meaningful contribution to the limited body of research on PTSD in under-resourced, post-conflict settings. The use of validated assessment tools enhances the reliability and contextual relevance of the results. In addition, the disaggregation of data by gender and symptom profiles offers a more nuanced understanding of trauma experiences within the study population, even though some differences observed were not statistically significant. Future research would benefit from adopting longitudinal or mixed-methods designs to explore the temporal and potentially causal relationships between trauma exposure, somatic symptoms, and contextual factors. Repeated-measures or cohort studies could provide deeper insights into the dynamic interactions between psychological distress and cultural frameworks of healing. Additionally, expanding the study population to include participants from diverse healthcare settings such as public, rural, and specialized clinics would strengthen the external validity and contextual relevance of the findings.

## 6 Conclusion

This study reveals a significant negative association between PTSD symptoms and body satisfaction, extending beyond psychological manifestations to include physical health complications. Patients with PTSD are markedly more likely to report conditions such as hypertension, diabetes, and cardiovascular symptoms, including tachycardia, palpitations, and respiratory issues. Despite relatively high awareness of clinical psychology services, the actual utilization of these services remains low. Instead, individuals with PTSD often turn to traditional and spiritual forms of support, reflecting culturally embedded care-seeking behaviours shaped by the local sociocultural context.

These results call for in-depth consideration of intervention and psychological support strategies in contexts where cultural norms and care practices are diverse. It appears necessary to develop integrative clinical approaches, sensitive to local representations of care and suffering, in order to promote greater accessibility and acceptability of mental health services emphasizing the need of interdisciplinary and intercultural work to adapt care systems to the realities on the ground, and thus better meet the needs of people suffering from PTSD in pluralistic contexts.

## Data Availability

The raw data supporting the conclusions of this article will be made available by the authors, without undue reservation.
